# Dimethyl 6,6′-dicyano-2,2′-bipyridine-3,3′-dicarboxyl­ate

**DOI:** 10.1107/S1600536809012446

**Published:** 2009-04-08

**Authors:** Xiao He, Gui-Rong Qu, Dongsheng Deng, Baoming Ji

**Affiliations:** a College of Chemistry and Environmental Science, Henan Normal University, Xinxiang 453007, People’s Republic of China; b College of Chemistry and Chemical Engineering, Luoyang Normal University, Luoyang 471022, People’s Republic of China

## Abstract

In the title compound, C_16_H_10_N_4_O_4_, the two pyridine rings are twisted by 44.41 (2)° and the ester groups form dihedral angles of 48.77 (4) and 45.75 (2)° with the corresponding pyridine rings. The crystal structure is stabilized by inter­molecular C—H⋯O hydrogen bonds and π–π stacking inter­actions between the pyridine rings [centroid-to-centroid distance 3.797 (2) Å].

## Related literature

For the synthetic procedures relevant to preparation of the title compound, see: Tichy *et al.* (1995 ▶); Glaup *et al.* (2005 ▶); Heirtzler (1999 ▶)
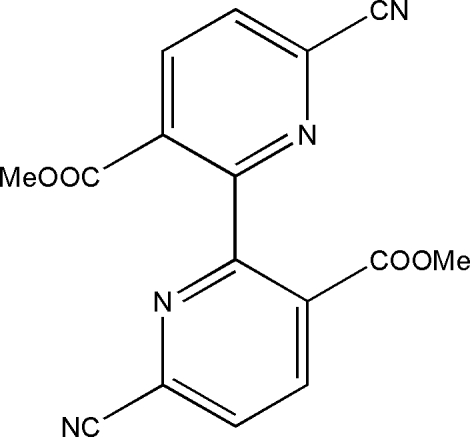


## Experimental

### 

#### Crystal data

C_16_H_10_N_4_O_4_
*M* *_r_* = 322.28
Triclinic, 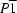

*a* = 8.201 (3) Å
*b* = 10.302 (6) Å
*c* = 10.768 (3) Å
α = 109.148 (4)°
β = 106.091 (3)°
γ = 100.404 (5)°
*V* = 787.9 (6) Å^3^
*Z* = 2
Mo *K*α radiation
μ = 0.10 mm^−1^
*T* = 296 K
0.49 × 0.45 × 0.41 mm

#### Data collection

Bruker APEXII CCD diffractometer
Absorption correction: multi-scan (*SADABS*; Sheldrick, 1996 ▶) *T*_min_ = 0.952, *T*_max_ = 0.960
5613 measured reflections
2845 independent reflections
2391 reflections with *I* > 2σ(*I*)
*R*_int_ = 0.014

#### Refinement

*R*[*F* ^2^ > 2σ(*F* ^2^)] = 0.034
*wR*(*F* ^2^) = 0.094
*S* = 1.05
2845 reflections
218 parameters
H-atom parameters constrained
Δρ_max_ = 0.15 e Å^−3^
Δρ_min_ = −0.12 e Å^−3^

### 

Data collection: *APEX2* (Bruker, 2004 ▶); cell refinement: *SAINT* (Bruker, 2004 ▶); data reduction: *SAINT*; program(s) used to solve structure: *SHELXS97* (Sheldrick, 2008 ▶); program(s) used to refine structure: *SHELXS97* (Sheldrick, 2008 ▶); molecular graphics: *SHELXTL* (Sheldrick, 2008 ▶) and *DIAMOND* (Brandenburg, 2006 ▶); software used to prepare material for publication: *SHELXTL* and *PLATON* (Spek, 2009 ▶).

## Supplementary Material

Crystal structure: contains datablocks global, I. DOI: 10.1107/S1600536809012446/gk2200sup1.cif

Structure factors: contains datablocks I. DOI: 10.1107/S1600536809012446/gk2200Isup2.hkl

Additional supplementary materials:  crystallographic information; 3D view; checkCIF report

## Figures and Tables

**Table 1:** Hydrogen-bond geometry (Å, °)

*D*—H⋯*A*	*D*—H	H⋯*A*	*D*⋯*A*	*D*—H⋯*A*
C4—H4*A*⋯O4^i^	0.93	2.39	3.222 (3)	149

Symmetry code: (i) 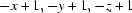.

**Fig. 1.**
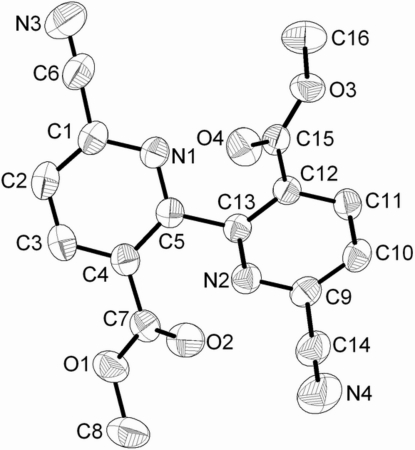
View of the title molecule with the atom numbering scheme and 30% probability displacement ellipsoids for non-hydrogen atoms. Hydrogen atoms are omitted for clarity.

**Fig. 2.**
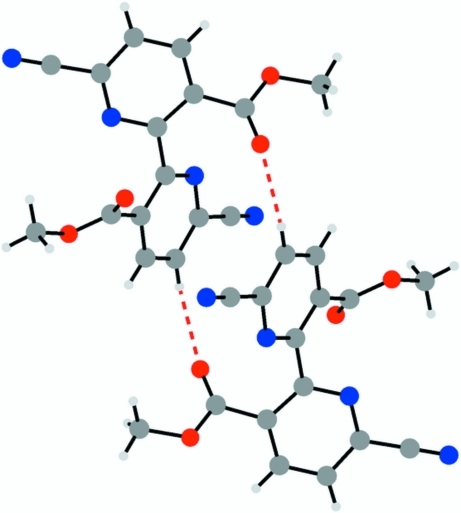
View of the centrosymmetric dimer; C—H···O hydrogen bonds are indicated with broken lines.

## supplementary crystallographic information

### Comment

Binicotinic acid and its derivatives have been proved to be a kind of multifunctional and flexible ligand in the construction of complexes possessing novel and interesting topological structures. Our interest in these compounds has led us to prepare the title compound. First, we synthesized dimethyl 2,2'-bipyridine-3,3'-dicarboxylate 1,1'-dioxide according to the reported method (Tichy *et al.* 1995). Second, the incorporation of cyano group onto 6 and 6' positions of the above compound could be readily performed when adopting the literature methods (Glaup *et al.* 2005; Heirtzler 1999). In this contribution, we report the synthesis and crystal structure of the title compound.

The asymmetric unit of the title compound contains one molecule (Fig. 1.). In the crystal structure, the most striking feature of the title compound is the interesting arrangement of the title molecules, which are linked into centrosymmetric dimers by formation of intermolecular C—H···O hydrogen bonds, in which C4—H4A is a donor and O4 is an acceptor (Table 1, Fig. 2). Short π···π contacts between two pyridine rings with centroid-centroid distance of 3.797 (2) Å are observed in the structure.

### Experimental

To an ice-cooled solution of dimethyl 2,2'-bipyridine-3,3'-dicarboxylate 1,1'-dioxide (1.22 g, 4 mmol) and trimethylsilyl cyanide (5.2 ml, 40 mmol) in *ca* 40 ml dry CH_2_Cl_2_ under N_2_ was carefully added benzoyl chloride (1.9 ml, 17 mmol). After stirring overnight at room temperature, 10% aq Na_2_CO_3_ was carefully added to the chilled reaction mixture and it was concentrated at 200 mbar to complete crude product precipitation. This was collected by filtration, washed with water and dried. Purification by silica gel chromatography using 100 ~200 mesh ZCX II eluted by hexane-ethyl acetate (3:1, *v*/*v*) gave the yellow solid. The crystalline compound was obtained by slow evaporation of CH_2_Cl_2_ solution containing the title compound.

### Refinement

All H atoms were positioned geometrically and treated as riding, with C—H bond lengths constrained to 0.93 Å (aromatic CH), 0.96 Å (methyl CH_3_), and with *U*ĩso~(H) = 1.2Ueq(C) or 1.5Ueq(methyl).

### Crystal data

**Table:**

C_16_H_10_N_4_O_4_	*Z* = 2
*M**_r_* = 322.28	*F*(000) = 332
Triclinic, *P*1	*D*_x_ = 1.358 Mg m^−^^3^
Hall symbol: -P 1	Mo *K*α radiation, λ = 0.71073 Å
*a* = 8.201 (3) Å	Cell parameters from 2697 reflections
*b* = 10.302 (6) Å	θ = 2.4–25.5°
*c* = 10.768 (3) Å	µ = 0.10 mm^−^^1^
α = 109.148 (4)°	*T* = 296 K
β = 106.091 (3)°	Block, yellow
γ = 100.404 (5)°	0.49 × 0.45 × 0.41 mm
*V* = 787.9 (6) Å^3^	

### Data collection

**Table:**

Bruker APEXII CCD diffractometer	2845 independent reflections
Radiation source: fine-focus sealed tube	2391 reflections with *I* > 2σ(*I*)
graphite	*R*_int_ = 0.014
Detector resolution: 0 pixels mm^-1^	θ_max_ = 25.5°, θ_min_ = 2.4°
phi and ω scans	*h* = −9→9
Absorption correction: multi-scan (*SADABS*; Sheldrick, 1996)	*k* = −12→12
*T*_min_ = 0.952, *T*_max_ = 0.960	*l* = −13→12
5613 measured reflections	

### Refinement

**Table:**

Refinement on *F*^2^	Secondary atom site location: difference Fourier map
Least-squares matrix: full	Hydrogen site location: inferred from neighbouring sites
*R*[*F*^2^ > 2σ(*F*^2^)] = 0.034	H-atom parameters constrained
*wR*(*F*^2^) = 0.094	*w* = 1/[σ^2^(*F*_o_^2^) + (0.0434*P*)^2^ + 0.140*P*] where *P* = (*F*_o_^2^ + 2*F*_c_^2^)/3
*S* = 1.05	(Δ/σ)_max_ < 0.001
2845 reflections	Δρ_max_ = 0.15 e Å^−^^3^
218 parameters	Δρ_min_ = −0.11 e Å^−^^3^
0 restraints	Extinction correction: *SHELXL*, Fc^*^=kFc[1+0.001xFc^2^λ^3^/sin(2θ)]^-1/4^
Primary atom site location: structure-invariant direct methods	Extinction coefficient: 0.044 (4)

### Special details

**Table:**

Geometry. All esds (except the esd in the dihedral angle between two l.s. planes) are estimated using the full covariance matrix. The cell esds are taken into account individually in the estimation of esds in distances, angles and torsion angles; correlations between esds in cell parameters are only used when they are defined by crystal symmetry. An approximate (isotropic) treatment of cell esds is used for estimating esds involving l.s. planes.
Refinement. Refinement of F^2^ against ALL reflections. The weighted R-factor wR and goodness of fit S are based on F^2^, conventional R-factors R are based on F, with F set to zero for negative F^2^. The threshold expression of F^2^ > 2sigma(F^2^) is used only for calculating R-factors(gt) etc. and is not relevant to the choice of reflections for refinement. R-factors based on F^2^ are statistically about twice as large as those based on F, and R- factors based on ALL data will be even larger.

### Fractional atomic coordinates and isotropic or equivalent isotropic displacement parameters (Å^2^)

**Table:**

	*x*	*y*	*z*	*U*_iso_*/*U*_eq_	
N1	0.24082 (14)	0.31796 (12)	0.50125 (12)	0.0430 (3)	
N2	0.10699 (15)	−0.04177 (12)	0.29318 (12)	0.0441 (3)	
N3	0.2207 (3)	0.64855 (17)	0.68504 (19)	0.0880 (5)	
N4	−0.1464 (3)	−0.37966 (17)	0.06250 (18)	0.0918 (6)	
O1	0.21123 (14)	0.07920 (12)	0.04429 (10)	0.0584 (3)	
O3	0.39330 (14)	0.18868 (11)	0.78250 (10)	0.0537 (3)	
O4	0.55513 (13)	0.24358 (11)	0.65966 (11)	0.0589 (3)	
O2	0.41072 (15)	0.03548 (12)	0.20273 (11)	0.0611 (3)	
C2	0.28278 (17)	0.22264 (15)	0.28064 (14)	0.0425 (3)	
C1	0.25460 (16)	0.20947 (14)	0.39931 (14)	0.0392 (3)	
C12	0.30974 (18)	0.10132 (15)	0.17406 (14)	0.0448 (3)	
C3	0.2918 (2)	0.35319 (16)	0.26803 (16)	0.0520 (4)	
H3A	0.3070	0.3642	0.1892	0.062*	
C4	0.2783 (2)	0.46654 (16)	0.37267 (17)	0.0534 (4)	
H4A	0.2850	0.5553	0.3668	0.064*	
C5	0.25452 (18)	0.44348 (14)	0.48638 (16)	0.0461 (3)	
C11	0.2373 (2)	0.55804 (17)	0.59939 (19)	0.0585 (4)	
C15	0.2181 (3)	−0.0415 (2)	−0.06874 (17)	0.0693 (5)	
H15A	0.1425	−0.0479	−0.1578	0.104*	
H15B	0.1782	−0.1288	−0.0572	0.104*	
H15C	0.3381	−0.0277	−0.0661	0.104*	
C9	0.10581 (18)	−0.19269 (14)	0.42201 (15)	0.0448 (3)	
H9A	0.0618	−0.2823	0.4223	0.054*	
C7	0.28339 (16)	0.05361 (13)	0.54006 (13)	0.0379 (3)	
C6	0.21790 (16)	0.06783 (13)	0.41195 (14)	0.0382 (3)	
C8	0.22261 (18)	−0.07911 (14)	0.54392 (15)	0.0432 (3)	
H8A	0.2603	−0.0914	0.6280	0.052*	
C10	0.05610 (17)	−0.16976 (14)	0.29983 (14)	0.0436 (3)	
C14	0.42572 (18)	0.17362 (14)	0.66546 (14)	0.0416 (3)	
C13	−0.0581 (2)	−0.28668 (17)	0.16696 (18)	0.0595 (4)	
C16	0.5312 (3)	0.2948 (2)	0.91195 (18)	0.0781 (6)	
H16D	0.4957	0.2978	0.9904	0.117*	
H16A	0.5497	0.3878	0.9077	0.117*	
H16B	0.6398	0.2692	0.9235	0.117*	

### Atomic displacement parameters (Å^2^)

**Table:**

	*U*^11^	*U*^22^	*U*^33^	*U*^12^	*U*^13^	*U*^23^
N1	0.0426 (6)	0.0382 (6)	0.0467 (7)	0.0097 (5)	0.0153 (5)	0.0172 (5)
N2	0.0445 (6)	0.0400 (6)	0.0427 (7)	0.0101 (5)	0.0113 (5)	0.0153 (5)
N3	0.1169 (15)	0.0572 (10)	0.0928 (13)	0.0325 (10)	0.0510 (11)	0.0193 (9)
N4	0.0980 (13)	0.0583 (10)	0.0702 (11)	0.0005 (9)	0.0005 (10)	0.0035 (9)
O1	0.0661 (7)	0.0712 (7)	0.0413 (6)	0.0318 (6)	0.0166 (5)	0.0232 (5)
O3	0.0612 (6)	0.0478 (6)	0.0391 (5)	0.0067 (5)	0.0151 (5)	0.0092 (5)
O4	0.0460 (6)	0.0583 (7)	0.0626 (7)	0.0006 (5)	0.0143 (5)	0.0246 (6)
O2	0.0594 (7)	0.0714 (7)	0.0530 (6)	0.0332 (6)	0.0153 (5)	0.0225 (6)
C2	0.0385 (7)	0.0450 (8)	0.0438 (8)	0.0108 (6)	0.0120 (6)	0.0209 (6)
C1	0.0361 (7)	0.0385 (7)	0.0406 (7)	0.0093 (5)	0.0106 (5)	0.0167 (6)
C12	0.0417 (7)	0.0506 (8)	0.0437 (8)	0.0114 (6)	0.0155 (6)	0.0220 (7)
C3	0.0543 (9)	0.0559 (9)	0.0567 (9)	0.0159 (7)	0.0229 (7)	0.0335 (8)
C4	0.0556 (9)	0.0434 (8)	0.0701 (10)	0.0160 (7)	0.0239 (8)	0.0317 (8)
C5	0.0422 (7)	0.0380 (7)	0.0571 (9)	0.0109 (6)	0.0168 (6)	0.0191 (7)
C11	0.0652 (10)	0.0415 (9)	0.0706 (11)	0.0165 (7)	0.0269 (8)	0.0225 (8)
C15	0.0746 (11)	0.0858 (13)	0.0416 (9)	0.0346 (10)	0.0174 (8)	0.0161 (9)
C9	0.0437 (7)	0.0360 (7)	0.0567 (9)	0.0096 (6)	0.0208 (6)	0.0200 (7)
C7	0.0376 (7)	0.0376 (7)	0.0402 (7)	0.0117 (5)	0.0162 (6)	0.0158 (6)
C6	0.0380 (7)	0.0369 (7)	0.0406 (7)	0.0111 (5)	0.0149 (6)	0.0159 (6)
C8	0.0467 (7)	0.0426 (8)	0.0448 (8)	0.0132 (6)	0.0183 (6)	0.0216 (6)
C10	0.0396 (7)	0.0370 (7)	0.0463 (8)	0.0083 (6)	0.0119 (6)	0.0118 (6)
C14	0.0433 (7)	0.0370 (7)	0.0442 (8)	0.0128 (6)	0.0135 (6)	0.0175 (6)
C13	0.0613 (10)	0.0434 (9)	0.0568 (10)	0.0070 (7)	0.0098 (8)	0.0136 (8)
C16	0.0843 (13)	0.0666 (11)	0.0445 (10)	0.0064 (10)	0.0046 (9)	−0.0007 (9)

### Geometric parameters (Å, °)

**Table:**

N1—C1	1.3328 (17)	C4—C5	1.378 (2)
N1—C5	1.3430 (18)	C4—H4A	0.9300
N2—C6	1.3322 (17)	C5—C11	1.451 (2)
N2—C10	1.3406 (18)	C15—H15A	0.9600
N3—C11	1.136 (2)	C15—H15B	0.9600
N4—C13	1.139 (2)	C15—H15C	0.9600
O1—C12	1.3252 (17)	C9—C10	1.376 (2)
O1—C15	1.4495 (19)	C9—C8	1.3786 (19)
O3—C14	1.3249 (17)	C9—H9A	0.9300
O3—C16	1.4483 (19)	C7—C8	1.3864 (19)
O4—C14	1.1999 (16)	C7—C6	1.4011 (18)
O2—C12	1.1997 (17)	C7—C14	1.4913 (19)
C2—C3	1.386 (2)	C8—H8A	0.9300
C2—C1	1.4038 (19)	C10—C13	1.447 (2)
C2—C12	1.492 (2)	C16—H16D	0.9600
C1—C6	1.4942 (19)	C16—H16A	0.9600
C3—C4	1.378 (2)	C16—H16B	0.9600
C3—H3A	0.9300		
			
C1—N1—C5	117.06 (12)	H15A—C15—H15C	109.5
C6—N2—C10	117.28 (12)	H15B—C15—H15C	109.5
C12—O1—C15	116.20 (12)	C10—C9—C8	118.15 (12)
C14—O3—C16	115.67 (13)	C10—C9—H9A	120.9
C3—C2—C1	118.21 (13)	C8—C9—H9A	120.9
C3—C2—C12	120.91 (12)	C8—C7—C6	118.08 (12)
C1—C2—C12	120.83 (12)	C8—C7—C14	120.67 (12)
N1—C1—C2	122.74 (12)	C6—C7—C14	121.05 (11)
N1—C1—C6	115.11 (11)	N2—C6—C7	122.92 (12)
C2—C1—C6	121.83 (12)	N2—C6—C1	114.08 (11)
O2—C12—O1	124.85 (14)	C7—C6—C1	122.83 (12)
O2—C12—C2	124.19 (13)	C9—C8—C7	119.38 (13)
O1—C12—C2	110.93 (12)	C9—C8—H8A	120.3
C4—C3—C2	119.75 (13)	C7—C8—H8A	120.3
C4—C3—H3A	120.1	N2—C10—C9	124.06 (12)
C2—C3—H3A	120.1	N2—C10—C13	115.18 (13)
C3—C4—C5	117.53 (13)	C9—C10—C13	120.76 (13)
C3—C4—H4A	121.2	O4—C14—O3	125.10 (13)
C5—C4—H4A	121.2	O4—C14—C7	123.42 (13)
N1—C5—C4	124.68 (13)	O3—C14—C7	111.43 (11)
N1—C5—C11	115.08 (13)	N4—C13—C10	179.1 (2)
C4—C5—C11	120.23 (13)	O3—C16—H16D	109.5
N3—C11—C5	178.05 (19)	O3—C16—H16A	109.5
O1—C15—H15A	109.5	H16D—C16—H16A	109.5
O1—C15—H15B	109.5	O3—C16—H16B	109.5
H15A—C15—H15B	109.5	H16D—C16—H16B	109.5
O1—C15—H15C	109.5	H16A—C16—H16B	109.5
			
C5—N1—C1—C2	0.20 (19)	C8—C7—C6—N2	−3.22 (19)
C5—N1—C1—C6	−173.30 (11)	C14—C7—C6—N2	171.65 (12)
C3—C2—C1—N1	−1.7 (2)	C8—C7—C6—C1	171.66 (11)
C12—C2—C1—N1	175.79 (12)	C14—C7—C6—C1	−13.47 (18)
C3—C2—C1—C6	171.35 (12)	N1—C1—C6—N2	132.04 (13)
C12—C2—C1—C6	−11.14 (19)	C2—C1—C6—N2	−41.53 (17)
C15—O1—C12—O2	5.1 (2)	N1—C1—C6—C7	−43.25 (17)
C15—O1—C12—C2	−176.63 (12)	C2—C1—C6—C7	143.18 (13)
C3—C2—C12—O2	129.19 (16)	C10—C9—C8—C7	0.99 (19)
C1—C2—C12—O2	−48.3 (2)	C6—C7—C8—C9	2.17 (19)
C3—C2—C12—O1	−49.07 (17)	C14—C7—C8—C9	−172.72 (12)
C1—C2—C12—O1	133.48 (13)	C6—N2—C10—C9	2.6 (2)
C1—C2—C3—C4	1.8 (2)	C6—N2—C10—C13	−177.41 (12)
C12—C2—C3—C4	−175.66 (13)	C8—C9—C10—N2	−3.6 (2)
C2—C3—C4—C5	−0.5 (2)	C8—C9—C10—C13	176.48 (13)
C1—N1—C5—C4	1.3 (2)	C16—O3—C14—O4	−2.7 (2)
C1—N1—C5—C11	179.56 (13)	C16—O3—C14—C7	175.13 (13)
C3—C4—C5—N1	−1.1 (2)	C8—C7—C14—O4	130.87 (15)
C3—C4—C5—C11	−179.31 (14)	C6—C7—C14—O4	−43.86 (19)
C10—N2—C6—C7	0.86 (19)	C8—C7—C14—O3	−46.97 (16)
C10—N2—C6—C1	−174.43 (11)	C6—C7—C14—O3	138.30 (12)

### Hydrogen-bond geometry (Å, °)

**Table:**

*D*—H···*A*	*D*—H	H···*A*	*D*···*A*	*D*—H···*A*
C4—H4A···O4^i^	0.93	2.39	3.222 (3)	149

Symmetry codes: (i) −*x*+1, −*y*+1, −*z*+1.
